# Income trajectories and self-rated health status in the UK

**DOI:** 10.1016/j.ssmph.2022.101035

**Published:** 2022-01-28

**Authors:** Lateef Akanni, Otto Lenhart, Alec Morton

**Affiliations:** a Department of Economics, University of Strathclyde Business School, 199 Cathedral Street, Glasgow, UK; b Department of Management Science, University of Strathclyde Business School, 199 Cathedral Street, Glasgow, UK

**Keywords:** Income trajectories, Self-rated health, Panel data, Fixed effects, United Kingdom

## Abstract

In line with the wide recognition of the connection between socioeconomic status and health outcomes, attention in the recent literature is extending the static perspective to the dynamic implications of income on health. This study contributes to the growing literature on the income-health nexus by evaluating income dynamics on various self-rated health measures in the UK. We explore the impact of different indicators of income experiences on self-rated health and wellbeing outcomes using data from the 11 Waves of Understanding Society UK Household Longitudinal Study between 2009 and 2019. First, we estimate a fixed-effects ordered logit model for various health and wellbeing measures, allowing us to control for unobserved time-invariant heterogeneity. Second, we evaluate the effects of income trajectories by linking longitudinal household income to cross-sectional health outcomes. Our results confirm the general evidence of positive impacts of increasing family income on health. Besides, we find that stability in income position is strongly associated with improved health and wellbeing. On the other hand, income volatility increases the odds of reporting poor health outcomes, particularly for those in low-income households. Also, more years spent in a lower-income quartile reduces the odds of reporting improved self-rated health. Finally, the significant difference in the estimated effects of income before and after 2016 highlights the significant shifts in the effects of income trajectories on self-reported health and wellbeing following the National Living Wage policy implementation.

## Highlights

• The paper evaluates the impact of income trajectories on health and wellbeing outcomes.
• Income stability is associated with increased likelihood of improved self-reported health.
• Income volatility and low-income spells predicts poor health and wellbeing.

## 1 Introduction

Anecdotal evidence suggests that poor socioeconomic status and poverty are connected to higher morbidity and mortality rates. However, there continued to be recurring issues when analysing the nexus between income and health. First, the empirical evidence is not conclusive on the causal relationship between income and health. While studies based on the cross-sectional analysis of income and health nexus are popular in the literature, their results are likely biased by confounding (Gunasekara, Carter, & Blakely, 2012). On the contrary, analysis of longitudinal data on income and health outcomes, which has been growing in popularity in recent literature, accounts for time-invariant confounding and provides better estimates of the health effects of income changes. Likewise, reversed causality and health selection can be reduced more efficiently using longitudinal approaches (Miething & Aberg Yngwe, 2014).

The second issue relates to the importance of income dynamics and its lagged effects on health and wellbeing. Although current income level and distribution are well-established causes of inequalities in the distribution of health and wellbeing status, there is supporting evidence that income measures based on more extended periods are better indicators of economic status (Benzeval & Judge, 2001). Moreover, both downward and upward trends in individual or household income are better predictors of health outcomes (Frech & Damaske, 2019; Schollgen, Kersten, & Rose, 2019). In addition, health and wellbeing are sometimes less sensitive to temporary fluctuations, including short periods of hardships, unemployment spells, and small changes in nominal incomes (Davillas, Jones, & Benzeval, 2019).

We contribute to the growing literature on income dynamics and health by revisiting the relationship between income and health using the 2009 to 2019 waves of the Understanding Society UK Household Longitudinal Survey (UKHLS). Given the ordinal nature of the health and wellbeing measures, we employ the fixed-effects ordered logit model (Baetschmann, Ballantyne, Staub, & Winkelmann, 2020; Ferrer‐I‐Carbonell & Frijters, 2004). Previous studies that employ fixed-effect models mainly split health responses into dichotomous variables to enable estimation using binary fixed effect models (see Benzeval & Judge, 2001; Collin et al., 2020). However, altering data distribution may affect central tendency and variance measures, which could also diminish the reliability and validity of the estimated results (Dell-Kuster et al., 2014). Additionally, accommodating the multi-item scale of health and wellbeing measures captures the sensitive changes in people's health conditions over time, as well as provides a complete profile of their health dimensions (Bowling, 2005). Secondly, other strand of studies employs ordinal outcomes following the random-effect model assumptions that the error term is normally distributed and independent of the regressors. However, the fixed-effect estimator controls for the presence of unobserved heterogeneity and time-invariant factors which might determine both income and health. Failure to account for these confounding factors could lead to biased results and spurious relationships (see Contoyannis, Jones, & Rice, 2004; Frijters, Haisken-DeNew, & Shields, 2005; Frijters & Ulker, 2008).

Furthermore, we evaluate the effects of stability and volatility in household income position and duration of low-income and high-income spells on health and wellbeing by combining longitudinal income dynamics with cross-sectional health and wellbeing data. Using the 18 Waves of the British Household Panel Survey (BHPS), Davillas et al. (2019) evaluate the long-run average measure of household income on self-rated health (SRH) and biomarkers. However, this study additionally considered the effects of income volatility, which reflects ongoing shifts in economic risks and threatens health and wellbeing (Prause, Dooley, & Huh, 2009). Additionally, we employed a novel measure of income volatility by dividing the change in income by the mean of current and immediate past income. The volatility measure ensures that the size of income change is not dependent on the ordering of incomes in either year. It also adjusts for outliers and the inclusion of observations where income is zero in both successive periods (see Avram, Brewer, Fisher, & Fumagalli, 2019). Besides, the larger sample size and comprehensive geographical coverage of the UKHLS data used in this study provide a more recent and broader scope of the income-health dynamics across the full-age range, ethnic groups, and socioeconomic diversity (Platt, Knies, Luthra, Nandi, & Benzeval, 2021).

We further consider how household position on the overall income distribution affects health and other wellbeing outcomes (see also Miething & Aberg Yngwe, 2014). In addition, we conduct separate analyses of the impact of income trajectories on health outcomes for low-income and high-income households. Thus, we contribute to literature providing information on health risks, especially those likely suffered by individuals from low-income households. Lastly, we partition the empirical analyses into two sub-sample periods, before and after 2016, coinciding with the introduction of the UK's National Living Wage (NLW) policy. Since income is known to causally determine health, after controlling for possible confounders (Gunasekara, Carter, & Blakely, 2011), it is essential to isolate the effects of such policies that could affect the income-health nexus. Hence, we conduct a pre-post analysis to gain insight into the likely impact of the increased wage floor on the income trajectory and health nexus.

In line with related literature, we consider the different array of health measures, including the SRH, mental health and subjective wellbeing outcomes, including satisfaction with leisure and life (see Apouey & Clark, 2015). First, combining physical and mental health with wellbeing indicators is more relevant to policy. The World Health Organisation declared that “there can be no physical health without mental health” (Kolappa, Henderson, & Kishore, 2013, p. 3). Besides, the report of the 2019 global burden of disease showed that mental health disorders, including depression, anxiety and conduct disorders, bipolar, schizophrenia, and autism spectrum disorders, eating disorders, and a host of other residual category of mental disorders, remained one of the top-ten causes of disease burden globally, with no evidence of reduction in its burden since 1990 (GBD 2019 Mental Disorders Collaborators, 2022). In the UK, the importance of mental health is evidently a major policy agenda, with all major political parties having mental health goals included as part of their manifestos (Allwood & Bell, 2019).

The rest of the paper is structured as follows: Section 2 reviews the literature on the nexus between income dynamics and health outcomes. Section 3 discusses the model and variables measurement, while Section 4 describes the data. Section 5 presents the results and discussions, and finally, Section 6 concludes the paper.

## 2 Literature review

There is a long-standing debate concerning the proxies and data sources for health outcomes and wellbeing in evaluating the effects of income on population health and well-being. Broad health measures have been considered in the literature, including pathological and clinical measures extracted mostly from individual medical records and administrative data and individual SRH collected through surveys. The choice of health measures is determined by collection costs, ethical considerations, and external validity. As a result, SRH measures are prominently used in empirical studies. One of its features is the combination of different aspects of health. However, SRH measures are also widely criticised as subjective measures, and that they may not give adequate and efficient assessments of the objective state of public health (Johnston, Propper, & Shields, 2009).

Nonetheless, subjective measures can provide accurate and efficient assessments of objective states of health (Cleary, 1997). For example, through the interviewer's interaction with the respondents, they can objectively assess a self-rated measure of physical function by asking whether an individual has sight and hearing difficulties. However, the accuracy can further be attributed to the experience of ill health and health problems (Simon, De Boer, Joung, Bosma, & Mackenbach, 2005).

The increased availability and popularity of longitudinal surveys have led to considerable growth in the empirical application of panel data approaches to investigate the relationship between income and health. Contrary to the approach by early literature, which largely provided cross-sectional analyses of people's static income and health experience, studies based on longitudinal data capture the variation and dynamics of income over time. Using repeated measures of income is less prone to error than using income information for a particular year (Miething & Aberg Yngwe, 2014). Longitudinal studies also address the potential measurement problems and reporting errors arising from using SRH and subjective wellbeing measures since they combine a series of health measures from similar individuals over time. In addition, every individual assigns their scales to health ranking when comparing current to prior health assessments (Lenhart, 2017).

Empirical studies have also combined longitudinal income data with cross-sectional health dimensions to investigate how income affects health and well-being outcomes over time. Most of these studies’ findings suggest that a longer-term income position is more relevant in predicting health and wellbeing than current income. This finding is relatable to the Permanent Income Hypotheses (Friedman, 1957). Long-term income and socioeconomic position are more relevant to health and may reflect cumulative disadvantage than transitory status (Davillas et al., 2019). Benzeval and Judge (2001) found that family income averaged over five years better predicts SRH in the subsequent year than current income in any given year. Miething and Aberg Yngwe (2014) evaluate the role of stability and variability in individual income position over time using Swedish survey data. Their findings confirm that changes in income status and the time dimensions of income are important for health status.

Vanzella-Yang and Veenstra (2021), using ten years average income of Canadian households between 2002 and 2011, finds that stable income is strongly associated with SRH. In addition, they report that spending more years at the bottom quintile of income distribution corresponds to increased odds of reporting poor or fair health by men and vice versa for women. Also, Davillas et al. (2019) evaluate income and health gradients in the UK using the combination of the BPHS and UKHLS survey data. Their findings also support the long-term relationship between income and health, which is larger than short-term cross-sectional measures.

Studies on income and health nexus based on longitudinal analyses are also prone to different measurement problems. Gunasekara et al. (2012) evaluate the limitations in using SRH, mostly ignored as bias sources in longitudinal analyses. The first limitation is regarded as “longitudinal validity or responsiveness” (Gunasekara et al., 2012, p. 1118), and it relates to SRH skewness and its accuracy in measuring health changes over time. One example is the SRH ceiling effect, which implies individuals who had previously rated their general health as excellent cannot provide a higher response category when they feel additional improvements in their health. Second, the corresponding changes in SRH and its underlying health status measurement accuracy. The third limitation is the reference group effects, where respondents adjust their SRH health responses to their perceived reference group. The authors further find that using the 36-Item Short-Form Survey (SF-36), defined as a more detailed measure of self-assessed current health status, is less affected by the longitudinal validity bias (Gunasekara et al., 2012). Overall, these limitations point to shortcomings in using SRH as the only index to capture all aspects of health, especially in longitudinal analyses. Moreover, studies like Au and Johnston (2014) found that the weak or non-significant effects of income on health using SRH co-exist significantly with other health domains, such as mental health and wellbeing (see also Mavaddat et al., 2011). Overall, no single measure captures all the dimensions of health and wellbeing; hence, it is essential to be clear and detailed in specific aspects of health being evaluated as different health dimensions may move in the opposite direction in response to income changes (Apouey & Clark, 2015).

Furthermore, changes in income that can be ascribed to changes in wage-related policies are significant predictors of health and wellbeing outcomes (Leigh, Leigh, & Du, 2019; Lenhart, 2019; Reeves, McKee, Mackenbach, Whitehead, & Stuckler, 2017). For example, introducing the National Minimum Wage (NMW) and the National Living Wage (NLW) policies in the UK and the subsequent annual uprating in wage floors has led to a continuous increase in nominal wage rates. The standard approach employed in empirical literature to evaluate such policies effects includes methods similar to natural experiments, particularly the Randomized Control Trials (Craig, Katikireddi, Leyland, & Popham, 2017). However, limited or no studies evaluate minimum wage policy using RCTs, given the costs and other ethical considerations for conducting such experiments. Instead, previous studies follow quasi-experimental approaches such as those that compare the health outcomes of individuals that received increased wages with a homogenous comparison group who do not receive the increase (Leigh, 2021).

Empirical studies have also adopted various estimation methods to investigate both intended and unintended consequences of wage policy interventions by considering the effects of shifts in the population's income distribution. Although, these approaches may include individuals who are not “directly” affected by the wage policy and might produce inexact policy estimates (Renson, Chung, & Lodge, 2020). However, limiting the evaluation to only the individuals who receive the minimum wage increase could also underestimate the policy's exact effects. Besides, policymakers are usually interested in the results of policy evaluations that produce encompassing estimates that quantify the changes after minimum wage policies are introduced or changed (see Renson et al., 2020). Additionally, individuals' perception about an increase in income received by others due to a change in wage policy can serve as an additional pathway that connects minimum wage to health and wellbeing (Lenhart, 2017). The group of individuals who are indirectly affected might include: (i) the self-employed who do not receive wages; (ii) the unemployed, who might be affected by the consequent reduction in jobs availability, and (iii) other workers who are earning at or above the minimum wage but might experience wage compression (Buszkiewicz, Hill, & Otten, 2021).

## 3 Model and methods

### 3.1 Income and health outcomes

The first aspect of our empirical analysis involves evaluating the relationship between income and the different aspects of health and wellbeing using longitudinal data. Specifically, we begin by estimating the impact of income on health and wellbeing measures using longitudinal data on household income and selected health and wellbeing outcomes. Our choice of using the fixed effects ordinal logit model is to account for any potential endogeneity stemming from time-invariant characteristics. Besides, the estimator is useful for estimating causal effects between income and health (Baetschmann et al., 2020; Ferrer‐I‐Carbonell & Frijters, 2004).^1^

The model is specified as:

[1] $$h_{it}^{\text{*}}=\alpha_{i}+y_{it}^{\prime }\beta +X_{it}^{\prime }\delta +e_{it}$$

[2] $$h_{it}=k\Leftrightarrow h_{it}^{\text{*}}\in \left[\varphi_{ik},\varphi_{ik+1}\right]$$

where $h_{it}^{\text{*}}$ is the latent health variable corresponding to the health outcome for individual *i*'s at time *t*, while h is the observed health and wellbeing measure. y indicates income measured as natural log of household after-tax and inflation-adjusted equivalized income. X is a set of observable time-varying control variables. The considered covariates include age, age-squared, age-cubed, education attainment, marital status, number of people employed in the household, and region of residence.

The parameters, β and δ are the coefficients of the main explanatory and control variables, respectively. Both indicate the direction in which an increase in the regressors impacts the cumulative distribution of the health outcome. An estimated β>0 and statistically significant indicates that an increase in household income will cause an increase in the probability of the highest health outcome category $\left[Pr\left(h_{it}\geq K|y_{it},\alpha_{i}\right)\right]$ and a decrease in the probability of the lowest category $\left[Pr\left(h_{it}\geq 1|y_{it},\alpha_{i}\right)\right]$. $\alpha_{i}$ indicates time-invariant, individual-specific fixed effects, and $e_{it}$ is the time-varying logit-distributed, orthogonal error term.

Equation [2] ties the latent outcome variable $h^{\text{*}}$ to the observed ordered health outcome variable, h through the threshold, $\varphi_{ik}$, where $\varphi_{ik}$ is the cut-off point, increasing in k, with k indicating each response category for the health outcomes. The probability of observing the health category k for individual i in period t, which also depends on household income (y) and parameter (β) is given as:

[3] $$Pr\left(h_{it}=k|y_{it},\alpha_{i}\right)=\Psi \left(\varphi_{ik+1}-y_{it}^{\prime }\beta -\alpha_{i}\right)-\Psi \left(\varphi_{ik}-y_{it}^{\prime }\beta -\alpha_{i}\right)$$

By extension, equation [3] indicates that the probability also depends on the individual-specific fixed effects ($\alpha_{i}$) and the cut-off point (φ). In addition to the direction of effects (β), we evaluate the size effect of household income on health outcomes using the odds ratio (OR). OR is the ratio between the probability of a certain outcome and the complementary probability (Baetschmann et al., 2020). Hence, the odds of an individual i in period t having a health outcome above category k relative to a lower or equal outcome to K is defined as:

[4] $$odds\left(k,y_{it}\right)\equiv \frac{Pr\left(h_{it}>k|y_{it}\right)}{Pr\left(h_{it}\leq k|y_{it}\right)}\equiv \exp\left(y_{it}^{\prime }\beta -\varphi_{ik}\right)$$

On the other hand, the changes in odds due to changes in the regressor, which depends both on the β and the regressor shift is given as:

[5] $$OR\left(k,\Delta y_{it}\right)=\frac{odds\left(k,y_{it}+\Delta y_{it}\right)}{odds\left(k,y_{it}\right)}=\exp\left(\Delta y_{it}^{\prime }\beta \right)$$

where OR is the odds ratio. It implies that a unit increase in household income increases the odds ratio by about (exp(β)−1)×100% for all categories of health outcomes except the first (see Baetschmann et al., 2020).

### 3.2 Income stability, volatility, and trajectory

In addition to evaluating the impact of household current on health and wellbeing using longitudinal data, we also assess the effects of income dynamics on health by pooling household income over cross-sectional health and wellbeing measures. Specifically, we consider various aspects of income trajectories on health outcomes including, *stability in income position*, calculated as the average of equivalized and inflation-adjusted household after-tax income. A similar approach has been employed in extant literature to assess the effects of income stability on both subjective and objective health outcomes (see Benzeval & Judge, 2001; Davillas et al., 2019; Frech & Damaske, 2019; Miething & Aberg Yngwe, 2014; Schollgen et al., 2019).

Secondly, we evaluate the impact of *income volatility* on health and wellbeing outcomes. There is no established consensus on measuring income volatility. The prominent approach used in literature is to measure income instability or volatility as the deviations of an individual or household's income from the average over a defined period (Prause et al., 2009; The Aspen Institute, 2016). We define income volatility as the standard deviation of the arc percentage change in income:

$$volatility_{it}=\sqrt{Variance\left[\left(\frac{y_{it}-y_{it-1}}{\left(y_{it}+y_{it-1}\right)/2}\right)\times 100\right]}$$

where $y_{it}$ indicates household real disposable income for individual *i* in period *t*. The division of the change in income by the mean of current and immediate past income ensures that the size of income change is not dependent on the ordering of incomes in either year. It also adjusts for outliers and the inclusion of observations where household income is zero in both successive periods. The measure has also been shown to be closely related to the variance of transitory shocks in income using more complex models (see also Avram et al., 2019). We rescale both the income stability and volatility measures to standardised logged values using zero mean and a standard deviation of one to facilitate results interpretation.

Furthermore, we filter out the health effects of changes in household income by separately evaluating the extent to which the health effects of income differ for those in low and high-income groups. Besides, the increase in “deaths of despair” arising mainly from drugs overdoses, alcohol, and suicides deaths have been ascribed to the stagnant and falling income levels and decreasing labour market opportunities (Allik, Brown, Dundas, & Leyland, 2020; Case & Deaton, 2017). Consequently, the third aspect of our analysis involves evaluating the health effects of enduring low-income or high-income spells. We create separate variables that consider the number of years household real disposable income was below the median or above the median income. Lastly, we evaluate the differential effects of income trajectories on health and wellbeing before and after 2016. The pre-post analysis provides insights into the intersection of income trajectories and health outcomes coinciding with the NLW implementation.

In addition to covariates including age, age-squared, age-cubed, marital status, number of people employed in the household, and region of residence, we include gender and ethnic group as an additional covariate in the cross-sectional income trajectory models. Gender difference has been widely acknowledged as a key determinant of the inequalities in health and wellbeing outcomes. In addition, the inclusion of individual's ethnicity as control is motivated by the strong association between race and reporting poorer self-reported health status (see Subramanian & Kawachi, 2003; Wolff, Subramanian, Acevedo-Garcia, Weber, & Kawachi, 2010).

### 3.3 Health and wellbeing outcomes measures

As discussed in the introduction, we considered different dimensions of health and wellbeing outcomes, including general and mental health, satisfaction with leisure, and life satisfaction. Health measures that are based on self-reports are shown to be reliable, valid, and comparable over multiple periods by different studies including Contoyannis et al. (2004), Vaillant and Wolff (2012), and Dasgupta (2018), among others. Also, Simon et al. (2005) shows that assessing general health status through SRH can encompass health dimensions beyond physical health, including mental and other health-related behaviours. However, SRH remains a health measure in the general population that remains poorly understood (Gunasekara et al., 2012). Therefore, using SRH as the only index to capture all aspects of health in the analysis of income and health relationship can lead to incorrect inference. For example, while some studies found income to have little or no effect on SRH, the non-significant effects of income on health outcome using SRH are found to co-exist significantly with other dimensions of health (see Au & Johnston, 2014). Hence, a separate evaluation of income effects on mental health outcomes will further provide a detailed analysis of the income-health nexus specific to cognitive health, including anxiety and depression, social dysfunction, and loss of confidence.

The prevalence of mental health problems and their measurement could be daunting due to the hidden nature of mental health issues and variations in diagnostic practices across countries. In addition, there are devolved mental health measures across different countries, making it difficult and sometimes impossible to comparatively determine the prevalence of mental health problems across countries because of differences in the methodological approach used in measurements. However, longitudinal data may provide reliable evaluation and statistically comparable estimates over time since similar methods and techniques are repeatedly adopted in mental health measurements (see Garcia & Marder, 2017). In addition, leisure satisfaction has been widely used in health studies as a core mediating or outcome variables (Biswas-Diener, 2008). Moreover, leisure satisfaction may unfold in various degrees during a person's life course, and consequently, may relate more or less strongly to health and overall life satisfaction (Gelissen, 2019, pp. 1–22). The measurement approach of the health and wellbeing outcomes considered are discussed as follow:

#### 3.3.1 General health

The health indicator used in empirical studies is usually based on how survey questions and responses are constructed. However, most surveys treat health outcomes using ordinal measures, with good health being better than poor health (Frijters & Ulker, 2008). The UKHLS collects information about participants’ health status. Specifically, it asked respondents to rate their general health condition over a five-scale from 1 indicating excellent health to 5, poor health status. However, we invert the responses by recoding 1 to indicate poor health and 5 indicating excellent health to facilitate consistency in interpreting the estimated results.

#### 3.3.2 Mental health

The General Health Questionnaire (GHQ-12) has been widely adopted in mental and health research as the validated screening tool for psychiatric illness (see Griffith & Jones, 2019; Kronenberg, Jacobs, & Zucchelli, 2017). Hu, Stewart-Brown, Twigg, and Weich (2007), using exploratory and confirmatory factor analyses of BHPS and Health Survey for England (HSE) data, confirmed that GHQ-12 consistently measures different dimensions of mental health in population-based research. The GHQ-12 questions comprise six positively worded and six negatively worded questions that describe respondents’ mood states over a few weeks before the interview (Brown, Harris, Srivastava, & Taylor, 2018). The sub-components of the GHQ-12 questions are recoded and summed to a single scale from 0 to 12. However, we reverse the caseness score to increase from 1 (most distressed) to 13 (least distressed) to measure mental health and easier results interpretation.

#### 3.3.3 Leisure and life satisfaction

We measure leisure and life satisfaction using the respective indicators from the UKHLS. The leisure satisfaction ranks respondents' satisfaction with the amount of leisure time. Similarly, life satisfaction is the raking of their overall life over a seven-scale from completely unsatisfied to completely satisfied. The extent to which people are satisfied with their leisure time and activities has been employed in the empirical literature, and it's an important predictor of their overall wellbeing. For example, Gelissen (2019, pp. 1–22) found satisfaction with leisure time a consistent indicator of overall leisure satisfaction.

## 4 Data and descriptive statistics

The data used for the empirical analysis are drawn from waves 1 to 11 of the UKHLS. The UKHLS is an annual survey of members of approximately 40,000 households. The households are selected across geographical areas of the four countries of the UK, including England, Scotland, Wales, and Northern Ireland. The selected households from the first wave were repeatedly followed over time, making the survey one of the largest longitudinal surveys of its kind. The study is built on the former British Household Panel Survey (BHPS), which ran between 1991 and 2009, by including the approximately 8000 households of the original BPHS. Also, it covers all age groups, and it is multi-topic, covering a range of social, economic, and behavioural factors. Also, it is designed with an Immigrant and Ethnic Minority Boost sample by allowing for increased sample sizes for different ethnic minority and immigrant groups. Buck and McFall (2011) and McFall, Nandi, and Platt (2017) provided a complete discussion on the survey design overview and sample structure.^2^

The quality and applicability of the UKHLS data in empirical and policy research is well-rooted and demonstrated in earlier research, including studies on income, health, and wellbeing (Avram et al., 2019; Davillas et al., 2019; Knies, 2017). Besides, the *UKHLS* has been described as “the data source for many research papers where income plays a central role, even if it is not the main outcome variable of the analysis” (Fisher, Fumagalli, Buck, & Avram, 2019, p. 3). Using UKHLS as the single data source allows the study of different health outcomes for the same individual over time, using the same controls. Besides, it eliminates the difference between estimates found in studies that use other data sources (see also Frijters & Ulker, 2008).

Using the UKHLS data also ensures a balanced cross-section of individuals over time-dimension. Data collection for each survey wave usually takes over 24 months, with the collection period for different successive waves overlapping, thus giving a complex data design. However, we collect data for all the variables of interest by pooling them correspondingly across different intersecting waves and harmonising them into uniquely identified calendar years to ensure that our analysis sample is nationally representative (see Kaminska & Lynn, 2019). Therefore, the calendar periods for empirical analysis after the harmonisation start and end in 2011 and 2019.

Following our main objective, we restrict the analysis to a sample of respondents with valid household income data. Lastly, as earlier discussed, we partitioned the analysis into periods before and after 2016. The summary of means and standard deviations for household income and the considered health outcomes are summarised in Table 1. The descriptive statistics show that the average real household disposable income increased with less deviation from the average for the 2016–2019 sub-sample, increasing real disposable income across households over time. However, the health and wellbeing indicators are comparably similar across the three sub-samples.

**Table 1:** Descriptive statistic: income and health outcomes.

	Full sample		2011–2015		2016–2019	
	Mean	Std. Dev.	Mean	Std. Dev.	Mean	Std. Dev.
Household disposable income	1890.70	1955.56	1882.17	2088.58	1901.09	1780.28
Health & wellbeing outcomes						
SRH	3.374	1.054	3.480	1.059	3.251	1.0338
GHQ-12	11.374	2.932	11.389	2.892	11.357	2.977
Leisure satisfaction	4.880	1.662	4.788	1.692	4.987	1.619
Life satisfaction	5.229	1.4314	5.218	1.445	5.243	1.415
No of cross-sections	16442		16103		17230	

**Note**: Household disposable income is the equivalized and inflation-adjusted household net income measured in British Pounds.

## 5 Results and discussions

### 5.1 The longitudinal fixed-effects model for health and wellbeing outcomes

Table 2 summarises the coefficients of the estimated fixed-effects model for the health and wellbeing indicators. The main explanatory variable is the log of household disposable income, inflation-adjusted and equivalized using the OECD-modified equivalence scale. The use of equivalized after-tax income is mainly to adjust for family size and composition (see also Miething & Aberg Yngwe, 2014). In addition, the log transformation of income accounts for the skewness in the income distribution. We considered different covariates, including age, age-squared, age-cubed, marital status, number of household members in employment, educational attainment, and region of residence.

**Table 2:** Fixed effects ordered logit model of health and wellbeing outcomes.

	SRH	GHQ-12	Leisure satisfaction	Life satisfaction
Household disposable income (log)	0.056***	0.150***	−0.067***	0.102***
	(0.016)	(0.019)	(0.016)	(0.016)
Age	−0.151***	−0.260***	−0.311***	−0.257***
	(0.029)	(0.031)	(0.026)	(0.029)
Age-squared	0.000	0.005***	0.007***	0.005***
	(0.001)	(0.001)	(0.001)	(0.001)
Age-cubed	−0.000	−0.000***	−0.000***	−0.000***
	(0.000)	(0.000)	(0.000)	(0.000)
Marital status				
***Ref:****Never married*				
Married or Cohabiting	−0.104*	0.221***	−0.032	0.359***
	(0.058)	(0.060)	(0.050)	(0.055)
Unmarried (widowed, divorced & Separated)	0.020	−0.053	0.131**	0.111*
	(0.067)	(0.069)	(0.059)	(0.063)
Education attainment				
***Ref:****No qualification*				
GCSE, A-level, etc	0.285	−0.367*	−0.319**	−0.203
	(0.173)	(0.206)	(0.152)	(0.161)
Other qualification	0.316	−0.064	−0.059	0.045
	(0.195)	(0.230)	(0.176)	(0.185)
Degree and other higher degree	0.354*	0.032	−0.222	0.047
	(0.204)	(0.241)	(0.186)	(0.199)
Number employed in HH	0.028**	0.057***	−0.160***	0.020
	(0.014)	(0.016)	(0.013)	(0.014)
Regional dummies	Yes	Yes	Yes	Yes
Observations	219679	695248	478978	388550

Note: ***, ** and * denotes statistical significance at 1%, 5% and 10% levels respectively. Standard errors are presented in parenthesis. Household disposable income (log) is the log of the equivalized and inflation-adjusted after-tax household income SRH denotes self-rated health, while GHQ-12 is the mental health indicator. The models are estimated using the fixed effects ordered logit model.

Household real disposable income positively affects general health, mental health, and life satisfaction, while the effect is negative for satisfaction with leisure time. The significant positive coefficients indicate that increase in household disposable real income increase the probability of reporting excellent SRH, the least distress in mental health outcome, and complete satisfaction with overall life. At the same time, it decreases the likelihood of reporting the lowest category for the three outcomes, respectively. An increase in household disposable income increases the odds ratio of reporting improved SRH for all categories from fair to excellent health status, by about 5.76% [(exp(0.056)−1)].

Additionally, the odds ratio for GHQ-12 is about 16.18% [(exp(0.150)−1)] to report less distress in mental health outcomes. In comparison, life satisfaction has an odds ratio of about 10.74% [(exp(0.102)−1)]. This indicates an increased likelihood of reporting improved satisfaction with overall life as household income increases. On the contrary, the coefficients of the log of household disposable income for the leisure satisfaction estimation is negative and significant, indicating a reduction in the likelihood of reporting improvement in leisure satisfaction as income increases.

The results further show that age and its cubed term both have negative and statistically significant coefficients for all the health and wellbeing outcomes, while age-squared is positive and significant, except for SRH. Thus, the results confirm the declining likelihood of reporting improved health and wellbeing as age increases. Besides, the results support findings in the literature on the non-linear relationship between age and health status (see Lorem, Schirmer, Wang, & Emaus, 2017; Moret et al., 2007). The estimated coefficients for marital status for the SRH is not statistically significant, indicating no significant difference in the likelihood of reporting changes in health and wellbeing status as an individual's marital status changes. Similarly, the significant coefficients for education attainment indicates an increased likelihood of reporting improved SRH as education attainment improves. On the other hand, the estimated results for the remaining three other outcomes, mental health, leisure and life satisfaction, show mixed signs and significance. Specifically, the nonsignificant coefficients indicate no significant difference in the likelihood of reporting improved health and wellbeing status as an individual's marital status or education attainment changes.

Finally, the number of household members in employment increases the likelihood of reporting improvements in general and mental health as well as life satisfaction but a decrease in the probability of reporting improved satisfaction with leisure. For improvement in SRH from poor to excellent, the compensating variation^3^ between having more members of the household that are working and an increase in household income is about 0.50 (0.028/0.056). The value implies that the log of household disposable income must increase by about 35.91% [exp(0.50)−1] to compensate for every unit reduction in the number employed in households for an individual's general health status to change from poor to excellent. On the contrary, the results show that a higher number of households members in employment has a negative and significant coefficient with leisure satisfaction.

### 5.2 Income trajectories and health outcomes

Next, we estimate cross-sectional regressions of health and wellbeing outcomes using longitudinal income information. The summary of the estimated coefficients and odds ratios for the different income gradients models across the health and wellbeing indicators are summarised in Table 3. The estimated models also control for age, including its squared and cubed terms, gender, marital status, education status, number of people employed in the household, ethnicity, and region of residence. The results summarised as Model I in Table 3 show that stability in household real disposable income position over the period under consideration is positive and significantly impacts all the health and wellbeing outcomes. The estimated coefficients and odds ratios show that average household income increases the likelihood of reporting better and improved general and mental health outcomes. In addition, stability in income position also increases the odds of reporting improved satisfaction with leisure time and overall life.

**Table 3:** Income – gradient and health outcome.

	*Income measure*	SRH	GHQ-12	Leisure	Life
*Model I*	Average household income	0.282***	0.122***	0.201***	0.280***
		[1.326]	[1.130]	[1.223]	[1.323]
		(0.020)	(0.023)	(0.020)	(0.020)
*Model II*	Income volatility	−0.007	−0.057***	−0.030*	−0.083***
		[0.993]	[0.944]	[0.970]	[0.920]
		(0.017)	(0.018)	(0.016)	(0.017)
*Model III*	Below median income	−0.066***	−0.032***	−0.047***	−0.050***
		[0.936]	[0.969]	[0.954]	[0.951]
		(0.006)	(0.007)	(0.006)	(0.006)
*Model IV*	Above median income	0.066***	0.032***	0.047***	0.050***
		[1.068]	[1.032]	[1.048]	[1.051]
		(0.007)	(0.007)	(0.007)	(0.007)

Note: ***, ** and * denotes statistical significance at 1%, 5% and 10% levels respectively. Odds ratios are presented in squared brackets, and standard errors are in parenthesis. SRH denotes self-rated health, while GHQ-12 is the mental health indicator. Leisure is satisfaction with leisure time, and life denotes satisfaction with overall life. All models are estimated using the ordered logit model. The covariates include age, age-squared, age-cubed, gender, marital status, the number employed in the household, ethnicity, and region of residence.

Model II summarises the estimated results for the second income gradient model, which evaluates the volatility in household income position on health and wellbeing. The estimated odds ratios show that the estimated coefficients of household income volatility are negative across the health and wellbeing outcomes but statistically significant for mental health and life satisfaction. The results suggest that increased volatility in household disposable real income decreases the likelihood of reporting improvements in health and wellbeing.

Models III and IV in Table 5 explored the length of time individuals endure low-income and high-income spells. Each variable comprises nine categories between never below (or above) the median income up to a maximum of eight periods below (or above) the median income.^4^ An increase in the number of years that household real disposable income is below the median income significantly reduces the odds ratio of reporting improved health and wellbeing. On the contrary, the increase in the length of time with family real disposable income above the median income increases the odds ratio for reporting excellent SRH, less distress in mental health, and complete satisfaction with leisure and life.

Overall, the results indicate that long-term income and stability in income position over time increases the likelihood of reporting improved physical and mental health and increased satisfaction with leisure and life. On the contrary, volatility in household income and household position on the general income distribution are significant predictors of health and wellbeing.

### 5.3 Partitioned analyses: low- and high-income households

**Fig. 1:**
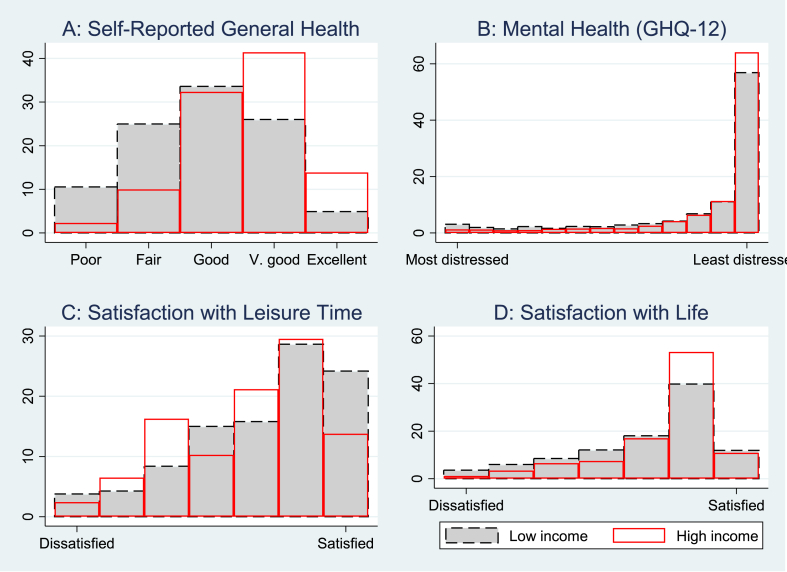
Distribution of health and wellbeing outcomes.

Additionally, we evaluate the variations in income gradients on health outcomes by separately estimating the health effects of income stability and volatility for individuals in low- and high-income households. The estimated results in the preceding section provide estimates of the expected variations in household income position and other covariates on the expected level of health outcomes across individuals irrespective of their income position in the distribution. However, the effect of income on health and wellbeing is not uniform throughout the distribution of income (Marmot, 2002). Besides, recent studies that extended the conventional regression approaches in evaluating the health effects of income using distributional regressions techniques found that households with poor income are particularly faced with more significant health risks. Also, they are at the lower end of the health distribution (see Kessels, Hoornweg, Thanh Bui, & Erreygers, 2020; Silbersdorff, Lynch, Klasen, & Kneib, 2018). Using the health and income distribution data in 2019, Fig. 1 (A - D) depicts distributions of health and wellbeing indicators for households in the bottom and top 20 percent of the equivalized net income distribution. The figures show substantial variations in health outcomes between households in the lower and upper part of the income distribution. For example, low-income households are skewed to have more risks of poor general health and most distressed mental health. Similar variations are noted for satisfaction with leisure time and overall life (see Fig. 1).

Consequently, we compare the health effects of stable and volatile income for individuals in low-income (bottom 20%) and high-income (top 20%) households.^5^ The results summarised in Table 4 show that the estimated coefficients and odds ratios for average household disposable income are positive across all the health and wellbeing outcomes for individuals in low-income households. However, it is statistically significant for general health and life satisfaction. In a similar vein, the estimated coefficients and odds ratios are positive for individuals in the high-income quartile. Again, it is statistically significant across all the health and wellbeing outcomes.

**Table 4:** Income trajectories and health outcome: low- and high-income households.

	SRH	GHQ-12	Leisure	Life
Bottom 20% Household				
Average household income	0.104**	0.059	0.136***	0.170***
	[1.109]	[1.061]	[1.146]	[1.186]
	(0.055)	(0.055)	(0.056)	(0.058)
Income volatility	0.002	−0.083**	−0.025	−0.085**
	[1.002]	[0.921]	[0.976]	[0.919]
	(0.032)	(0.033)	(0.034)	(0.031)
***Top 20% Household***				
Average household income	0.360***	0.107*	0.178***	0.313***
	[1.433]	[1.113]	[1.195]	[1.367]
	(0.070)	(0.061)	(0.061)	(0.068)
Income volatility	0.070*	0.037	0.075**	−0.016
	[1.073]	[1.038]	[1.078]	[0.984]
	(0.043)	(0.042)	(0.040)	(0.039)

Note: ***, ** and * denotes statistical significance at 1%, 5% and 10% levels respectively. Odds ratios are presented in squared brackets, and standard errors are in parenthesis. SRH denotes self-rated health, while GHQ-12 is the mental health indicator. Leisure is satisfaction with leisure time, and life denotes satisfaction with overall life. All models are estimated using the ordered logit model. The covariates include age, age-squared, age-cubed, gender, marital status, the number employed in the household, ethnicity, and region of residence.

**Table 5:** Income trajectories and health: before and after 2016.

	*Income measure*	SRH		GHQ-12		Leisure		Life	
		Before	After	Before	After	Before	After	Before	After
***Baseline***	Household disposable income (*log*)	0.025	0.058**	0.115***	0.128***	−0.072***	−0.068***	0.106***	0.038
		(0.025)	(0.027)	(0.030)	(0.029)	(0.024)	(0.025)	(0.026)	(0.026)
***Model I***	Average household income	0.287***	0.241***	0.106***	0.091***	0.160***	0.159***	0.239***	0.245***
		(0.019)	(0.020)	(0.022)	(0.022)	(0.019)	(0.021)	(0.020)	(0.020)
***Model II***	Income volatility	0.008	−0.050***	−0.037**	−0.083***	0.009	−0.061***	−0.033**	−0.114***
		(0.016)	(0.017)	(0.018)	(0.019)	(0.016)	(0.017)	(0.016)	(0.017)
***Model III***	Below median income	−0.110***	−0.104***	−0.036***	−0.050***	−0.069***	−0.083***	−0.083***	−0.086***
		(0.009)	(0.011)	(0.010)	(0.012)	(0.009)	(0.011)	(0.010)	(0.012)
***Model IV***	Above median income	0.110***	0.104***	0.036***	0.050***	0.069***	0.083***	0.083***	0.086***
		(0.009)	(0.011)	(0.010)	(0.012)	(0.009)	(0.011)	(0.010)	(0.012)

Note: ***, ** and * denotes statistical significance at 1%, 5% and 10% levels respectively. Standard errors are in parenthesis. *Before* implies estimation using data sample before the NLW policy, between 2011 and 2015, and *After* denotes estimated results using the 2016–2019 sample. SRH denotes self-rated health, while GHQ-12 is the mental health indicator. Leisure is satisfaction with leisure time, and life denotes satisfaction with overall life. The Baseline result is estimated using the fixed-effects ordered logit model, while Models 1 – IV are estimated using the cross-sectional ordered logit models. The covariates include age, age-squared, age-cubed, marital status, the number employed in the household, and region of residence. Models 1 – IV include gender and ethnicity as additional covariates.

On the other hand, income volatility is positive but not statistically significant for low-income households, while it is negative across all the other health and wellbeing outcomes but statistically significant for mental health and life satisfaction. For the high-income quartile, the estimated results are positive for all outcomes, except for life satisfaction, which is not statistically significant. Overall, the results suggest that income stability is vital for health and wellbeing irrespective of the household's income or position on the income distribution ladder. In contrast, volatility in household disposable real income significantly affects low-income households. Volatile income is associated with the increasing likelihood of reporting poor health and wellbeing outcomes.

### 5.4 Sub-sample analysis: before and after 2016

In this section, we discuss the results of the sub-sample estimation before and after implementing the NLW policy. While the results do not provide any causal estimates of the policy, it provides insights into the likely changes in size and magnitude of income effects on health and wellbeing outcomes. Therefore, we focus the discussion mainly on the estimates of the log of household disposable income and the various income trajectory indicators considered in the previous analysis, before and after 2016. The results are summarised in Table 5.

The estimated results using the fixed-effect models are summarised as the baseline model in Table 5. Current household disposable income is significantly associated with all the health and wellbeing outcomes considered, except for SRH and life satisfaction in the pre-2016 and post-2016 results respectively. Summarily, the non-significance for SRH estimation using the pre-2016 sample suggests that household income exerts a stronger effect on self-reported health in the periods following the NLW policy. On the other hand, the life satisfaction results may suggest that increased household income is associated with a declining trend in life satisfaction.

Furthermore, Models I to IV show the estimated coefficients of the different income trajectories on health and wellbeing outcomes. Similar to the estimated results using the entire data sample (Table 3), the estimated coefficients for average income are significant across all the health and wellbeing indicators, both before and after NLW. In contrast, the estimated coefficients of income volatility in Model II are significant in the post-2016 period (see Table 5).

To statistically evaluate the differences in the income effects on the health and wellbeing outcomes between the sub-sample periods, we compare the odds ratios of income trajectories on the various health and wellbeing outcomes before and after 2016.^6^ The plausibility of comparing estimates between logistic regressions models for different sub-samples using some of the popular methods available for linear regression models has been well discussed in the literature (see Allison, 1999; Kuha & Mills, 2020; Mood, 2010; Williams, 2009). Therefore, we followed the procedure proposed in Buis (2015) by evaluating the difference in odds ratios in the pre- and post-2016 estimation for each income trajectory variable: income stability, income volatility, length of time above and below the median income (see also Kuha & Mills, 2020). The computed Wald tests are summarised in Table 6. The results show that the predicted differential in the odds ratios is statistically significant across the models. By implication, the results suggest that there are statistically significant differences in the estimated odds ratios of the income trajectory indicators before and after 2016. Overall, the results suggest significant shift in the income trajectories effects on health and wellbeing outcomes, before and after implementing the NLW policy.

**Table 6:** Wald tests of the exponentiated combination of coefficients, before and after 2016.

		SRH	GHQ-12	Leisure	Life
***Model I***	Average household income	0.791*** (0.016)	0.923*** (0.019)	0.837*** (0.017)	0.780*** (0.016)
***Model II***	Income volatility	1.018* (0.011)	1.044*** (0.012)	1.032*** (0.011)	1.066*** (0.011)
***Model III***	Below median income	1.114*** (0.015)	1.048*** (0.015)	1.088*** (0.015)	1.081*** (0.015)
***Model IV***	Above median income	0.898*** (0.012)	0.954*** (0.014)	0.919*** (0.013)	0.925*** (0.013)

Note: ***, ** and * denotes statistical significance at 1%, 5% and 10% levels respectively. standard errors are reported in parenthesis. SRH denotes self-rated health, while GHQ-12 is the mental health indicator. Leisure is satisfaction with leisure time, and life denotes satisfaction with overall life.

## 6 Conclusion

This study contributes to the growing literature on income and health relationship by evaluating how income dynamics affects various subjective health and wellbeing outcomes. We explore different aspects of income experience on health and wellbeing using data from the Understanding Society's UK Household Longitudinal Study between 2009 and 2019. In addition to fully exploiting the longitudinal dimension of the data, our empirical approach accounts for the time-invariant endogeneity in income–health relationships. We also explore the health implications of changing income trajectories, including stability and variability in income position and duration of spells in low and high income.

The estimated fixed effects model results show that increased household income is associated with an increased likelihood of reporting excellent general health outcome, the least distress in mental health outcomes, and more satisfaction with life. These results are comparable to Davillas et al. (2019) who found that individuals who reported excellent health had higher household incomes than those likely to report lower SRH category. Analogous to the findings for life satisfaction, Knies (2017) previously found that UK households with higher family income have members who are more satisfied with their life than those in less income households. However, the negative coefficients for leisure satisfaction corroborates the labour supply decisions theory which assumed trade-off between income and leisure (see also Chadi & Hetschko, 2017; Powdthavee, 2012).

Additional results from the cross-sectional regressions show that income trajectories and time dimension matter for self-reported health and wellbeing. We find that stability in household disposable income is positive and significantly associated with the considered health and wellbeing outcomes. We also find that the length of time individuals endure low (high) income reduces (increases) their odds ratio of reporting improved self-rated health and subjective wellbeing. On the other hand, the negative coefficients for income volatility coupled with their position on the income distribution may suggest that unstable household income is associated with a declining trend in health and wellbeing. This could further be related to the submission in Wilson (2020) that observed increased wages in the UK had not reflected in comparably changes in price levels with income post-tax and national insurance contribution making people feel poorer at the end of every month (the Office of National Statistics [ONS], 2018). Concurrently, there is a continuous increase in “people's concerns about the general economic outlook and decreased real household spending per person (see also ONS, 2020).

Lastly, the sub-sample analyses using the sample partitioned into periods before and after the NLW policy implementation show significant results. Thus, it suggests a significant shift in the effects of income trajectories on health and wellbeing following the NLW policy implementation. Nonetheless, these findings do not provide the actual causal effects of the NLW policy effect on the relationship between income dynamics and health outcomes. One of the implications of these findings is that policies designed to address health and wellbeing problems must consider income volatility as an important source of risks, in addition to existing socio-economic factors. The significance of volatile incomes especially for low-income households further suggest that existing social policies and safety net programmes designed targeting low income individuals must also be designed with strengthened measures that deals with rising income volatility.

## Authorship statement

All persons who meet authorship criteria are listed as authors, and all authors certify that they have participated sufficiently in the work to take public responsibility for the content of the manuscript. Furthermore, each author certifies that this material or similar material has not been and will not be submitted to or published in any other publication before its appearance in the Social Science and Medicine – Population Health The list of the authors and their contributions are provided below: **Lateef Akanni**: Conceptualisation, Methodology, Formal Analysis, Writing – Original Draft, Writing – Review & Editing, **Otto Lenhart**: Supervision, Resources, Funding Acquisition, Writing – Review & Editing, **Alec Morton**: Supervision, Resources, Funding Acquisition, Writing – Review & Editing.

## Footnotes

1 The model is an extension of the Chamberlain (1980) fixed-effects binary logit model. Hence, for dichotomous health outcomes, the model approximates Chamberlain model. Also, to the best of our knowledge, very few studies have applied panel data fixed effects models on income-health nexus using ordinal health outcomes (see Carman, 2013; Frijters et al., 2005; Frijters & Ulker, 2008; Gunasekara et al., 2012).

2 See Institute for Social and Economic Research (2020) for a complete user guide of the UKHLS survey.

3 The compensating variation is computed as the ratio of the corresponding coefficients of the regressors (see Baetschmann et al., 2020).

4 Annual median income is computed separately for each calendar year between the 2011 and 2019 (see also Miething & Aberg Yngwe, 2014).

5 The classification of households into low and high income is based on the distribution of income using 2018 equivalized household monthly disposable income.

6 The procedure followed in computing the Wald tests is only compatible with cross-sectional, non-clustered standard errors. However, the fixed-effects ordinal logit model employed in the baseline model (Table 5) requires fitting the model with clustered standard errors. More importantly, using the estimator without clusters can be misleading (see Baetschmann et al., 2020, p. 270). Hence, we report the Wald tests results for Models 1 – IV.
